# Improving effect of soy whey‐derived peptide on the quality characteristics of functional yogurt

**DOI:** 10.1002/fsn3.3312

**Published:** 2023-03-13

**Authors:** Fatemeh Mashayekh, Rezvan Pourahmad, Mohammad Reza Eshaghi, Behrouz Akbari‐Adergani

**Affiliations:** ^1^ Department of Food Science and Technology Varamin‐ Pishva Branch, Islamic Azad University Varamin Iran; ^2^ Water Safety Research Center Food and Drug Administration, Ministry of Health and Medical Education Tehran Iran

**Keywords:** bioactive peptide, functional, quality characteristics, soy whey, yogurt

## Abstract

The purpose of this research was to investigate the effect of bioactive peptides isolated from soy whey on the physicochemical, sensory, and microbiological characteristics of yogurt during storage. Trypsin was utilized to hydrolyze soy whey protein at 45°C for 4 h. Then, the resulting protein hydrolysate was fractionated using reversed phase‐high performance liquid chromatography (RP‐HPLC). Since the F7 fraction showed the best antioxidant and antibacterial capabilities, different levels (6.5, 13, and 17 mg/mL) of this peptide fraction were added to yogurt. A control sample (without the bioactive peptide) was also prepared. Yogurt samples were stored for 3 weeks. With the increase in peptide concentration, the antioxidant activity of yogurt increased while viscosity and syneresis decreased (*p* < .05). During storage, yogurt acidity, syneresis, and viscosity increased while pH and antioxidant activity declined (*p* < .05). The addition of bioactive peptide reduced the quantity of *Escherichia coli* and *Staphylococcus aureus* bacteria in yogurt during storage (*p* < .05), and the reduction in bacterial quantity was stronger as the peptide content was increased. The sample containing the largest concentration of peptide (17 mg/mL) got the lowest overall acceptability score. The level of 13 mg/mL of the peptide was chosen as the best concentration for yogurt fortification in terms of overall acceptance and functional properties. Therefore, soy whey‐derived peptide can be utilized as a functional component as well as a natural preservative in yogurt.

## Graphical abstract

Since soy whey is a cost‐effective source of peptides and little research has been done on peptides derived from this by‐product, the aim of this study was to isolate and purify bioactive peptides from this by‐product and use them as a natural preservative in a model food system (yogurt). The fraction with the highest antimicrobial and antioxidant activity which was added to yogurt showed a good antibacterial effect against *Escheirichia coli* and *Staphylococcus aureus*. Moreover, the addition of this peptide improved the physicochemical quality of yogurt. Therefore, this bioactive peptide can be used as a natural alternative to common preservatives in the food industry.

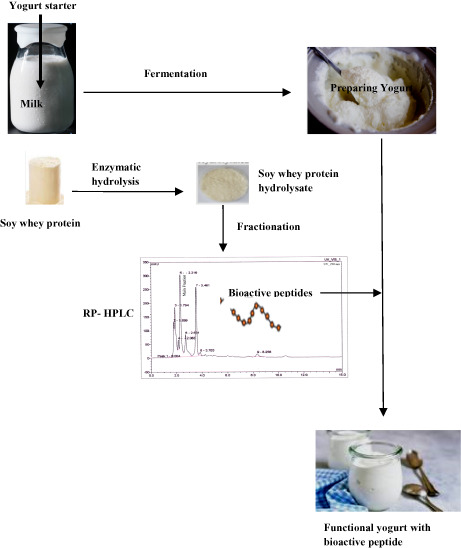

## 1 INTRODUCTION

In the recent two decades, research on bioactive peptides has exploded, and several studies have been undertaken in this sector. Recent scientific research reveals that dietary proteins may affect the body's physiological activities in addition to acting as nutrients. Bioactive peptides are chemical compounds made up of amino acids that are linked by covalent connections known as amide or peptide bonds. Proteins are polypeptides with a greater molecular weight. Bioactive peptides and proteins play a vital part in living organisms' metabolic activities and, as a result, in human health. The overwhelming majority of bioactive peptides are encoded in protein structure and released mostly by enzymatic activities. Chemical synthesis has been used to create several bioactive peptides. Bioactive peptides influence the gastrointestinal tract, endocrine, cardiovascular, immunological, and neurological systems, and hence play a significant role in human health (Altomare et al., 2020; Karami & Akbari‐Adergani, 2019; Wang et al., 2019). Bioactive peptides may help prevent oxidative spoilage of food as well as enhance the treatment of a variety of illnesses and disorders, improving the overall quality of life. A database named Biopep now contains over 1500 bioactive peptides (Mohan et al., 2019; Sánchez & Vásquez, 2017; Shaik & Sarbon, 2020; Singh et al., 2014). Antimicrobial peptides may be produced in two ways: by hydrolyzing dietary proteins or by bacterial secondary metabolites (bacteriocins). The antibacterial activity of these peptides has a variety of mechanisms of action. Although the majority of them affect the integrity of the target cell membrane by causing a hole in the cytoplasmic membrane, a few peptides target intracellular molecules and disrupt protein synthesis, enzyme function, or the cell wall (Farzaneh et al., 2019; Nicolas, 2009). Since antimicrobial peptides have a net positive charge and also exhibit hydrophobicity, they can combine with the negatively charged surface due to electrostatic interactions, penetrate and destroy the membrane, thereby killing the microorganisms (Wei & Zhang, 2022). Enzymatic breakdown of dietary proteins produces peptides with antioxidant properties (Chang et al., 2013; Kim et al., 2019; Liao et al., 2020; Waili et al., 2021; Wong et al., 2020). The antioxidant activity of peptides is therefore regulated by the amino acid content and order, which is influenced by the protein supply, hydrolysis conditions such as digesting enzyme, temperature, pH, reaction duration, and substrate‐to‐enzyme ratio (Aleman et al., 2011). Antioxidant activity of peptides is carried out through different mechanisms, including radical scavenging, metal chelation, electron or hydrogen transfer reduction, and aldehyde quenching (Durand et al., 2021).

Yogurt is a fermented milk product that is susceptible to microbial infection due to its high nutritional content, particularly at room temperature, which alters the product's flavor and fragrance and lowers its quality over time. This is a significant concern in the dairy sector, thus using natural and suitable antimicrobial agents to preserve quality and extend the shelf life of yogurt is critical. The addition of bioactive peptides to yogurt and dairy products has been studied. The impact of adding bioactive peptides derived from yogurt whey to doogh was explored by some researchers who found that the quantity of inoculated harmful bacteria drops dramatically during storage (Karimi et al., 2021). Oliveiralima et al. (2021) studied the impact of adding fish protein hydrolysate to yogurt and found that the antioxidant activity of the yogurt was boosted, as well as the product's physicochemical attributes.

Soy whey is a by‐product of tofu (soy cheese) production that contains a variety of nutrients such as proteins, oligosaccharides, minerals, and isoflavones (Hang, 2004; Yong‐Chua & Shao‐Quan, 2019). Soy whey proteins can be a good source of bioactive peptides. Some researchers investigated the effect of acylation with saturated fatty acids on the surface functional properties of tofu whey‐derived peptides. They found that chemical modification with fatty acids can influence the surface functional properties (emulsifying properties, surface hydrophobicity, fluorescence intensity, and water‐ and oil‐binding capacities) of these peptides, although the influence is dependent on the source of the peptide and degree of modification (Matemu et al., 2012). Since soy whey is a cost‐effective source of peptides and little research has been done on peptides derived from this by‐product, the purpose of this research was to isolate, purify, and use these peptides as a natural preservative in a model food system (yogurt) and also to investigate the effect of peptides derived from soy whey on the physicochemical, sensory, and microbiological characteristics of yogurt during storage.

## 2 MATERIALS AND METHODS

### 2.1 Preparation of soy whey protein hydrolysate

Soy whey was isolated from soy cheese produced using the method of Chumchuere et al. (2000). The pH change technique and whey pH adjustment in the range of 4.60 ± 0.05 were utilized to isolate soy whey protein. For this, 1 M hydrochloric acid was utilized. The acidified sample was then agitated for 2 min using a vortex stirrer. To separate the soy whey proteins, a chilled centrifuge (Model 30–30 Ks Hettich, Germany) was employed, and a sufficient volume of the acid‐treated whey sample was spun at 5800 rpm for 60 min. The top solution part of the sample was drained by overflowing the sample, and the residue at the bottom of the centrifuge tube contained soy whey protein. At pH = 7.8, a suitable quantity of extracted protein was dissolved in 50 mM phosphate buffer. Then, a solution of 6 mg/mL protein is generated by dissolving 600 mg of soy whey protein in distilled water and then increasing its volume in a 100 mL volumetric flask. The enzyme‐to‐substrate ratio of 1–12.5 was chosen for hydrolysis. In a shaker device at 45°C, the protein solution generated in the previous stage was continuously stirred at 125 rpm for 4 h. The trypsin enzyme was inactivated at 90°C for 15 min at the end of each stage, and the resultant solution was separated by centrifugation (10,000 × *g*) for 15 min. The top liquid created in this stage was placed in a falcon tube and kept at −18°C in the freezer. The sample was then transferred to a freeze drier to create a lyophilized version.

### 2.2 Isolation and purification of bioactive peptides

Bioactive peptides were isolated and purified using RP‐HPLC. For this, 40 mg of the frozen solution's extract was dissolved in 1 mL of solution A in a test tube. In this solution, there was 10% acetonitrile and 0.05% trifluoroacetic acid TFA in water. The test tube was centrifuged at 14,000 × *g* for 10 min after dissolution and then passed through a 0.45 μm filter. A 250‐L sample was injected into a C18 column (250 21.2 mm, 10 m) in an HPLC device (D‐14163 KNAUER, Knauer Germany). To remove the sample from the column, Solution B was applied. There was 0.05% TFA in 60% acetonitrile and 49.95% water in this solution. During the estimated test duration of 100 min, a linear gradient of 0–80% of solution B was administered. The absorbance of the samples was measured using a UV detector at a wavelength of 220 nm (Abadia‐Garcia et al., 2013; Pownall et al., 2010).

### 2.3 Antioxidant activity of peptide fractions

The DPPH reference technique was used to determine the antioxidant activity of each of the examined fractions. This procedure included mixing 500 μL of DPPH ethanol solution (0.2 Mm) with 1500 μL of the examined fraction's ethanol solution and storing the mixture in a dark spot at room temperature for 30 min. The solution adsorption was then measured using a spectrophotometer at 517 nm. The control sample was produced under the same circumstances as the test sample. With the exception that instead of the fraction solution, distilled water was utilized. This solution's adsorption was also measured at 517 nm (Liu et al., 2008). The following equation was used to determine and report the percentage of DPPH radical scavenging of each of the peptide fractions:

$$\text{The percentage of DPPH radical scavenging}={\left(A_{\text{control}}–A_{\text{sample}}/A_{\text{control}}\right)}^{*}\;100$$

where A_control_ is the control solution absorption and A_sample_ is the peptide fraction solution absorption.

### 2.4 Antimicrobial activity of peptide fractions

The antimicrobial activity of peptides against *Escherichia coli* PTCC 25922, and *Staphylococcus aureus* ATCC 25923 were evaluated by disk diffusion method. Each bacterium (1.5 * 10^8^ CFU/mL) was put onto plates with Brain Heart Infusion Agar. Lyophilized powders of these peptides were dissolved in water and the final peptide level was determined to be 1 mg/mL. For each bacterium, 20 μL of each peptide solution (100 mg/mL) was added to the paper disks in the plates, and each plate was incubated for 24 h at 37°C. The diameter of the growth inhibition zone was measured (Motta & Brandelli, 2002).

Minimum inhibitory concentration (MIC) and minimum bactericidal concentration (MBC) of the bioactive peptide against *E. coli* and *S. aureus* were determined by microdilution technique as described by Saei‐Dehkordi et al. (2010).

### 2.5 Production of yogurt

Milk (2.5% fat, 8.6% solids non‐fat) was pasteurized for 15 min at 85°C. Following this, the milk temperature was raised to 45°C and DVS yogurt starter (Chr. Hansen Co., Denmark) including *Streptococcus thermophilus* and *Lactobacillus delbrueckii* subsp. *bulgaricus* were added to the milk. At the inoculation time, the starter bacteria population was 10^8^ CFU/mL. Then the incubation was done until the pH reached 4.6, and finally, the product was cooled down until 10°C. *E. coli* and *S. aureus* were each added at a rate of 10^5^ CFU/mL. Moreover, yogurt was mixed with three different doses of bioactive peptide (6.5, 13, and 17 mg/mL peptide). A control sample was also made (without the bioactive peptide). The samples were kept at 4°C for 21 days. On days 1, 11, and 21, microbial features (*S. aureus* and *E. coli* counts) were evaluated. On days 1, 11, and 21, the physicochemical and sensory parameters of yogurt samples that were not infected with bacteria (*S. aureus* and *E. coli*) were also investigated.

### 2.6 Physicochemical analysis of yogurt

A pH meter (MP220 Model, Telodo Germany) was used to test the pH. Titatable acidity was determined according to AOAC method (AOAC, 2005). To determine syneresis, 15 g of yogurt samples were weighed into centrifuge tubes and spun for 30 min at 4°C at 3500 rpm in a centrifuge apparatus. After that, the supernatant was separated and weighed. The syneresis was calculated using the supernantant weight ratio to the weight of the initial yogurt (Ein Ali Afjeh et al., 2019).

A Brookfield viscometer (DVE type Brook field) was used to determine the viscosity of the generated samples. All tests were carried out for 40 s at a rotating speed of 20 rpm at a temperature of 20°C (Sah et al., 2016).

### 2.7 Yogurt antioxidant activity

Antioxidant activity was measured based on the DPPH method as described in Section 2.3.

### 2.8 Microbial properties of yogurt

To count *S. aureus*, 1 mL of yogurt sample was cultured on a plate containing Baird Parker Agar (Merck, Germany) and incubated for 24 h at 37°C (Anonymous, 2005).

To count *E. coli*, 1 mL of the sample was cultured on a plate containing MacConkie Agar (Merck, Germany) and incubated for 48 h at 37°C (Anonymous, 2015).

### 2.9 Sensory evaluation

Sensory features of yogurt samples without inoculated microorganisms (*E. coli* and *S. aureus*) were assessed by a panel of 12 trained members (6 men and 6 women, age range 20–45 years). The ranking method was used. The attributes (taste, odor, texture, color, and overall acceptance) were ranked with numbers 5, 4, 3, 2, and 1 equal to the very good, good, moderate, bad, and very bad degree of acceptability, respectively (Pourahmad & Mazaheri Assadi, 2007).

### 2.10 Statistical analysis

A one‐way analysis of variance was used to examine the findings. Duncan's multiple range test was used to compare the means at a 5% probability level. All experiments were performed in triplicate. The software utilized was SPSS 22.0.

## 3 RESULTS AND DISCUSSION

### 3.1 Isolation of peptide fractions by RP‐HPLC

**FIGURE 1:**
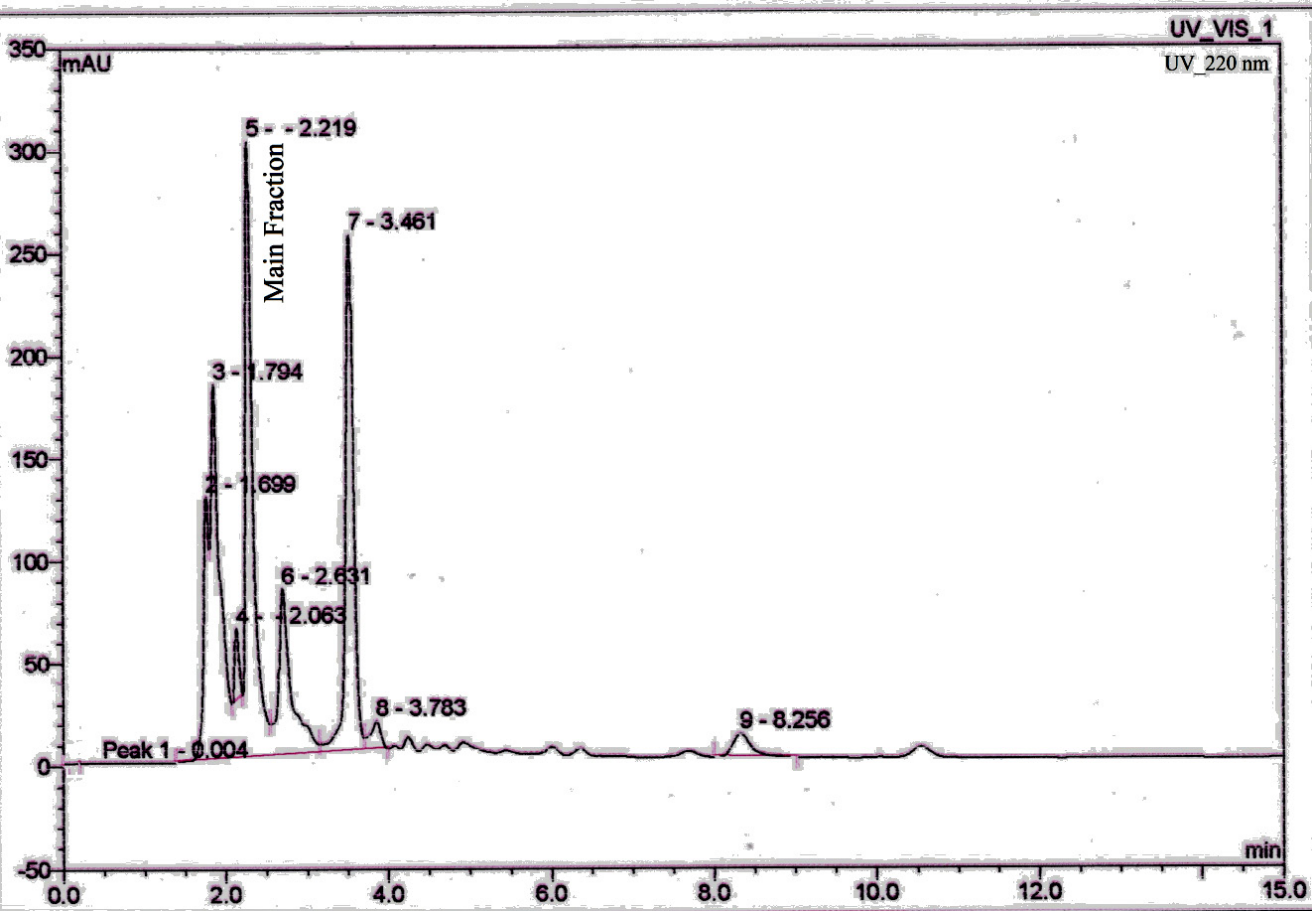
RP‐HPLC chromatogram of peptide fractions isolated from soy whey.

The chromatogram of peptide fractions generated from soy whey is shown in Figure 1. Nine peaks were obtained in the chromatogram and their antioxidant and antibacterial characteristics were examined. Table 1 shows that the F7 fraction has the best antioxidant and antibacterial properties, hence it was deemed the better fraction.

**TABLE 1:** Antioxidant and antimicrobial activity of peptide fractions isolated from soy whey (mean **±** standard deviation).

Fraction	IC_50_ (μg/mL)	Percentage of DPPH radical scavenging	Diameter of growth inhibition zone against *Escherichia coli* (mm)	Diameter of growth inhibition zone against *staphylococcus aureus* (mm)
F1	156.73 ± 0.50^e^	37.10 ± 0.30^c^	6.00 ± 1.00^b^	11.00 ± 1.00^c^
F2	145.83 ± 0.40^d^	38.80 ± 0.20^d^	6.00 ± 0.00^b^	12.00 ± 1.00^d^
F3	130.85 ± 0.50^c^	41.30 ± 0.30^e^	6.00 ± 1.00^b^	12.00 ± 1.00^d^
F4	130.81 ± 0.51^c^	42.80 ± 0.20^f^	5.00 ± 0.00^a^	10.00 ± 1.00^b^
F5	88.75 ± 0.25^b^	60.30 ± 0.30^g^	5.00 ± 0.00^a^	12.00 ± 0.00^d^
F7	70.01 ± 0.50^a^	64.90 ± 0.30^h^	7.00 ± 1.00^c^	15.00 ± 1.00^e^
F8	225.12 ± 0.41^g^	21.10 ± 0.10^a^	5.00 ± 0.00^a^	9.00 ± 1.00^a^
F9	188.41 ± 0.60^f^	27.50 ± 0.50^b^	5.00 ± 0.00^a^	9.00 ± 0.00^a^

*Note*: Dissimilar small letters indicate a significant difference in the column (*p* < .05).

The MIC of this fraction against *E. coli* and *S. aureus* was 13 and 2.1 mg/mL, respectively. The MBC of this fraction against *E. coli* and *S. aureus* was determined to be 17 and 4.5 mg/mL, respectively. Based on the results of MIC and MBC, three concentrations of 6.5 mg/mL (1/2 × MIC), 13 mg/mL (MIC), and 17 mg/mL (MBC) of this peptide fraction were added to yogurt. The physicochemical, microbial, and sensory characteristics of yogurt samples were investigated during storage.

### 3.2 Physicochemical properties of yogurt samples during storage

Table 2 shows that adding bioactive peptides to yogurt induces a substantial rise in pH during storage compared with control yogurt (*p* < .05). The explanation for this is that when the microbial growth rate in the treatments decreases, the pH rises more due to the generation of less acidic compounds by yogurt starter bacteria (Aryana & McGrew, 2007). According to Table 2, there was no significant difference in the pH of the samples on the first day of storage. On the eleventh and twenty‐first days, the highest pH value was associated with treatment 4 (yogurt containing 17 mg/mL of peptide), which was significantly (*p* < .05) different from the other treatments except 3 (yogurt containing 13 mg/mL of peptide), and the lowest pH value was associated with treatment 1 (control), which was significantly different from the other treatments except 2 (yogurt containing 6.5 mg/mL of peptide). In addition, from the first to the twenty‐first day, the pH of all samples reduced considerably (*p* < .05). According to Table 2, there was no significant difference in the acidity of samples on the first day of storage. On the eleventh day, the highest level of acidity was associated with treatment 1 (control), which did not differ significantly from the other treatments except 4 (yogurt containing 17 mg/mL of peptide), and the lowest level of acidity was associated with treatment 4 (yogurt containing 17 mg/mL of peptide), which did not differ significantly from the other treatments except 1 (control). Treatment 1 (control) had the greatest acidity on the 21st day, which was statistically (*p* < .05) different from the other treatments except 2 (yogurt with 6.5 mg/mL of peptide). Treatment 4 (yogurt containing 17 mg/mL of peptide) had the lowest level of acidity which was substantially (*p* < .05) different from the other treatments except 3 (yogurt containing 13 mg/mL of peptide). From the first to the twenty‐first day, the acidity of treatments 1 (control) and 2 (yogurt containing 6.5 mg/mL of peptide) rose considerably (*p* < .05). The findings show that adding bioactive peptides to yogurt substantially (*p* < .05) raises the acidity of all treatments over time. This is because, as storage time passes and starter bacteria continue to ferment lactose, acidity rises due to the formation of acids such lactic acid, formic acid, and acetic acid. In a similar study, Fang and Mingruo (2019) found that the acidity of yogurt samples containing hydrolyzed whey protein was lower than the control sample during 10 days of storage and that the pH of yogurt samples containing hydrolyzed whey protein was higher than the control sample. The influence of fish protein hydrolysate on the physicochemical parameters of stirred yogurt was examined by Oliveiralima et al. (2021). The pH of yogurt samples declined and acidity rose during 7 days of storage, which is consistent with the findings of this research. In a similar investigation, Samadi Varedesara et al. (2021) found that yogurt samples with grape seed protein hydrolysate had lower acidity and higher pH during 15 days of storage. According to Table 2, the largest syneresis was associated with treatment 1 (control) on the first, eleventh, and twenty‐first days of storage, while the lowest syneresis was associated with treatment 4 (yogurt containing 17 mg/mL of bioactive peptide). From the first to the twenty‐first day of storage, the rate of syneresis in all the samples rose considerably (*p* < .05). The findings show that adding bioactive peptides to yogurt reduces syneresis significantly (*p* < .05) compared with control yogurt. The lower syneresis of the peptides‐supplemented sample might be associated with the higher pH/lower acidity. The findings revealed that the quantity of syneresis increased with time, which might be attributable to an increase in acidity. Syneresis in yogurt is also caused by changes in the protein network's structure, which limits the capacity of proteins to bind to water (Aryana & McGrew, 2007; Ein Ali Afjeh et al., 2019; Sahan et al., 2008; Tamime et al., 1989). In a similar investigation, Bierzunska et al. (2019) found that the syneresis of yogurt samples containing hydrolyzed whey protein is lower than the control sample during 21 days of storage. Similarly, Oliveiralima et al. (2021) found that yogurt samples with hydrolyzed fish protein had less syneresis than the control sample during 7 days of storage. According to Table 2, on the first day of storage, the highest viscosity was associated with treatment 1 (control) and the lowest viscosity was associated with treatment 4 (yogurt containing 17 mg/mL of peptide), both of which were significantly (*p* < .05) different from the other treatments. Treatments 1 (control) and 4 (yogurt with 17 mg/mL of peptide) had the greatest and lowest viscosities on the eleventh and twenty‐first days of storage, respectively. The findings of the research show that adding bioactive peptides to yogurt reduces viscosity significantly (*p* < .05) compared with control yogurt. This drop may be due to the decrease in the activity of the yogurt starter, which produces lactic acid, lowers pH, and traps free water (Aryana & McGrew, 2007). Based on Table 2, the viscosity of all samples from the first to the twenty‐first day has a significant increase (*p* < .05). This is due to the proteins and casein micelles in yogurt trapping free water, which increases viscosity. This alteration might be caused by additional covalent and hydrogen bonding, higher hydration of the chemicals, increased water absorption, and the formation of stronger texture during storage in our three‐dimensional framework. In other words, increasing the viscosity of protein rearrangements and forming more protein–protein linkages are the major factors (Aryana & McGrew, 2007). Bierzunska et al. (2019) found that the viscosity of yogurt samples containing hydrolyzed whey protein rises during 21 days of storage, which is compatible with the findings of this study. Similarly, Oliveiralima et al. (2021) found that the viscosity of yogurt samples with hydrolyzed fish protein rises during 7 days of storage. Moreover, Samadi Varedesara et al. (2021) found that the viscosity of yogurt samples containing hydrolyzed grape seed protein increased during 15 days of storage, which is similar to the findings of this study.

**TABLE 2:** Physicochemical properties of yogurt samples during storage (mean **±** standard deviation).

Parameters	Samples	First day	11th day	21st day
pH	T1	4.52 ± 0.02^Ca^	4.41 ± 0.01^Ba^	4.27 ± 0.02^Aa^
	T2	4.53 ± 0.01^Ca^	4.42 ± 0.02^Bab^	4.32 ± 0.01^Ab^
	T3	4.54 ± 0.01^Ca^	4.44 ± 0.01^Bbc^	4.37 ± 0.02^Ac^
	T4	4.54 ± 0.02^Ca^	4.46 ± 0.01^Bc^	4.39 ± 0.02^Ac^
Acidity (percentage in terms of lactic acid)	T1	1.02 ± 0.02^Aa^	1.14 ± 0.03^Bb^	1.22 ± 0.03^Cc^
	T2	1.03 ± 0.03^Aa^	1.11 ± 0.04^Bab^	1.19 ± 0.02^Cbc^
	T3	1.04 ± 0.04^Aa^	1.08 ± 0.02^ABab^	1.14 ± 0.03^Bab^
	T4	1.03 ± 0.02^Aa^	1.06 ± 0.04^ABa^	1.12 ± 0.03^Ba^
Syneresis (%)	T1	18.50 ± 0.05^Ad^	23.94 ± 0.06^Bd^	28.87 ± 0.07^Cd^
	T2	17.41 ± 0.06^Ac^	20.03 ± 0.05^Bc^	21.48 ± 0.04^Cc^
	T3	15.23 ± 0.03^Ab^	18.32 ± 0.04^Bb^	19.84 ± 0.05^Cb^
	T4	14.59 ± 0.04^Aa^	17.57 ± 0.03^Ba^	18.74 ± 0.05^Ca^
Viscosity (cp)	T1	5.94 ± 0.05^Ac^	8.33 ± 0.03^Bd^	9.64 ± 0.04^Cd^
	T2	5.73 ± 0.03^Ab^	7.82 ± 0.05^Bc^	8.34 ± 0.04^Cc^
	T3	5.31 ± 0.06^Aa^	7.25 ± 0.05^Bb^	8.04 ± 0.04^Cb^
	T4	5.22 ± 0.05^Aa^	7.09 ± 0.03^Ba^	7.88 ± 0.04^Ca^

*Note*: T1: control, T2: yogurt containing 6.5 mg/mL of peptide, T3: yogurt containing 13 mg/mL of peptide, T4: yogurt containing 17 mg/mL of peptide.

Dissimilar capital letters indicate a significant difference in the row (*p* < .05). Dissimilar small letters indicate a significant difference in the column (*p* < .05).

### 3.3 Antioxidant activity of yogurt samples during storage

Treatment 4 (yogurt containing 17 mg/mL of peptide) had the greatest percentage of DPPH radical scavenging and the lowest IC_50_ on the first, eleventh, and twenty‐first days of storage, according to Table 3. Sample 1 (control) had the lowest percentage of radical scavenging and the greatest IC_50_. During storage, the percentage of DPPH radical scavenging reduced considerably in all samples, whereas IC_50_ rose (*p* < .05). The trapping of free radicals is the fundamental mode of action of antioxidants, which peptides use in the food system. The amino acid content, sequence, structure, and physicochemical activity of amino acids all influence the antioxidant activity of proteins and peptides (Power et al., 2013). The content and sequence of amino acids, as well as their structure, seem to alter following 21 days of storage due to the drop in pH and syneresis of yogurt, and it no longer has the original cohesiveness, lowering antioxidant activity. Karimi et al. (2022) studied the impact of adding bioactive peptides produced from yogurt whey on the antioxidant activity of doogh (Iranian fermented dairy drink), finding that as the content of bioactive peptides increases, so does the antioxidant activity of doogh. Doogh's antioxidant activity is also diminished during storage. These researchers' conclusions are in line with the findings of the current study. In another study, Samadi Varedesara et al. (2021) showed that the antioxidant activity of stirred yogurt samples containing hydrolyzed grape seed protein rises during 15 days of storage; however, our current study's findings are contradictory. Moreover, in a study on the influence of bioactive peptides produced from fish collagen on the characteristics of yogurt, some researchers indicated that antioxidant activity rises over the storage period of yogurt samples, however, the current research is contrary (Shori et al., 2021).

**TABLE 3:** Antioxidant activity of yogurt samples during storage (mean ± standard deviation).

Parameters	Samples	First day	11th day	21st day
IC_50_ (μg/mL)	T1	45.70 ± 0.40^Ad^	47.90 ± 0.50^Bd^	50.30 ± 0.60^Cd^
	T2	38.57 ± 0.38^Ac^	43.83 ± 0.59^Bc^	46.13 ± 0.51^Cc^
	T3	30.48 ± 0.29^Ab^	31.11 ± 0.61^Ab^	35.12 ± 0.69^Bb^
	T4	26.82 ± 0.39^Aa^	29.12 ± 0.31^Ba^	32.75 ± 0.49^Ca^
Percentage of DPPH radical scavenging	T1	7.29 ± 0.06^Ca^	6.11 ± 0.03^Ba^	5.42 ± 0.04^Aa^
	T2	8.13 ± 0.05^Cb^	6.90 ± 0.04^Bb^	6.25 ± 0.06^Ab^
	T3	12.55 ± 0.05^Cc^	11.05 ± 0.03^Bc^	9.32 ± 0.04^Ac^
	T4	14.84 ± 0.04^Cd^	13.28 ± 0.06^Bd^	12.44 ± 0.03^Ad^

*Note*: T1: control, T2: yogurt containing 6.5 mg/mL of peptide, T3: yogurt containing 13 mg/mL of peptide, T4: yogurt containing 17 mg/mL of peptide.

Dissimilar capital letters indicate a significant difference in the row (*p* < .05). Dissimilar small letters indicate a significant difference in the column (*p* < .05).

### 3.4 Microbial properties of yogurt samples during storage

According to Table 4, on the first, eleventh, and twenty‐first days of storage, the lowest and highest counts of *E. coli* were associated with treatments 4 (yogurt containing 17 mg/mL of peptide) and 1 (control), respectively; in fact, we can say that increasing the peptide concentration decreases the population of *E. coli*. As shown in Table 4, on the first, eleventh, and twenty‐first days of storage, treatments 4 (yogurt containing 17 mg/mL of peptide) and 1 (control) had the lowest and highest counts of *S. aureus*, respectively. The number of *S. aureus* reduces with increasing peptide concentration. Regarding the antimicrobial action of bioactive peptides, it should be emphasized that, by generating amphipathic structures on the surface of microbial membranes, these peptides induce membrane penetration and malfunction, so demonstrating their antimicrobial activity (Nicolas, 2009). Also, the number of *E. coli* and *S. aureus* bacteria in all treatments from the first to the twenty‐first day decreased significantly (*p* < .05) due to the accumulation of acid caused by the production of lactic acid and other organic acids, such as acetic acid, formic acid, etc., by yogurt starter bacteria, which results in a decrease in pH and an increase in acidity. In addition, raising the redox oxidation potential and the quantity of hydrogen peroxide produced by bacterial metabolism are factors in lowering the bacterial population in yogurt during storage (Ghaleh Mosiyani et al., 2017). Aguilar Toala et al. (2017) examined the multifunctional activity of bioactive peptides generated from fermented milk by *Lactobacillus plantarum* strains. During the 15‐day storage period of fermented milk samples, the bacterial count of *E. coli*, *Salmonella typhimurium*, and *Listeria innocua* reduced, corroborating the findings of this investigation. In a similar research, some researchers evaluated the usage of salal berry and black currant in yogurt to generate anti‐diabetic drinks. To isolate and identify peptides, reversed phase‐high performance liquid chromatography was paired with mass spectrometry. The quantity of *E. coli* and *Bacillus cereus* was greatly decreased by isolated peptides (Ni et al., 2018).

**TABLE 4:** Microbial properties of yogurt samples during storage (mean ± standard deviation).

Parameters	Samples	First day	11th day	21st day
*Escherichia coli* count (log CFU/mL)	T1	5.42 ± 0.012^Cd^	4.91 ± 0.004^Bd^	4.08 ± 0.050^Ad^
	T2	4.99 ± 0.002^Cc^	3.91 ± 0.012^Bc^	3.20 ± 0.109^Ac^
	T3	4.67 ± 0.006^Cb^	3.52 ± 0.037^Bb^	2.82 ± 0.000^Ab^
	T4	4.021 ± 0.008^Ca^	1.98 ± 0.006^Ba^	0.25 ± 0.241^Aa^
*Staphylococcus aureus* count (log CFU/mL)	T1	5.39 ± 0.016^Cd^	4.10 ± 0.015^Bd^	3.06 ± 0.033^Ad^
	T2	4.90 ± 0.001^Cc^	1.85 ± 0.015^Bc^	1.20 ± 0.109^Ac^
	T3	4.59 ± 0.007^Cb^	1.36 ± 0.065^Bb^	0.79 ± 0.102^Ab^
	T4	4.10 ± 0.040^Ca^	0.81 ± 0.105^Ba^	0.100 ± 0.173^Aa^

*Note*: T1: control, T2: yogurt containing 6.5 mg /mL of peptide, T3: yogurt containing 13 mg /mL of peptide, T4: yogurt containing 17 mg/mL of peptide.

Dissimilar capital letters indicate a significant difference in the row (*p* < .05). Dissimilar small letters indicate a significant difference in the column (*p* < .05).

### 3.5 Sensory properties of yogurt samples during storage

**FIGURE 2:**
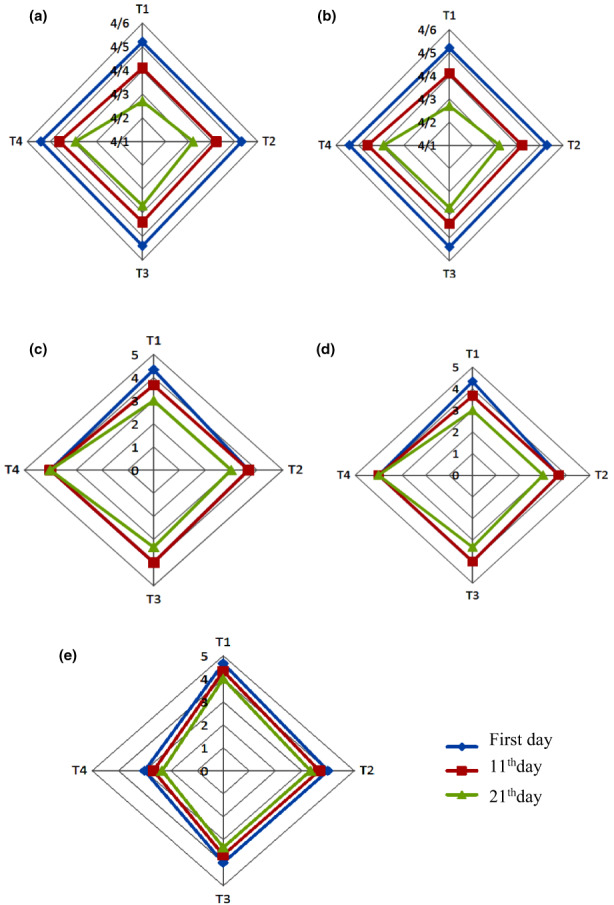
Sensory features scores of yogurt samples during storage. a: Taste; b: Smell; c: Texture; d: Color; e: Overall acceptabolity. T1: control, T2: yogurt containing 6.5 mg mL of peptide, T3: yogurt containing 13 mg/mL of peptide, T4: yogurt containing 17 mg/mL of peptide.

According to Figure 2, during storage, treatment 1 (control) had the highest taste sensory assessment score, and the lowest taste score was related to treatment 4 (yogurt containing 17 mg/mL of peptide). Also, the taste points of treatments 2 (yogurt containing 6.5 mg/mL of peptide), 1 (control), and 3 (yogurt containing 13 mg/mL of peptide) were not significantly different from day 1 to day 21. Due to the bitter taste of peptides, the addition of bioactive peptides to yogurt actually decreases the taste points substantially (*p* < .05) compared with control yogurt. Figure 2 demonstrates that from the first to the twenty‐first day, the taste rating of treatment 4 (yogurt containing 17 mg/mL of peptide) declined considerably (*p* < .05). Changes in yogurt's physicochemical and rheological qualities result in a shift in viscosity and a corresponding alteration in oral sensation, which contributes to the steady decline in taste scores. In addition, raising the acidity and decreasing the pH of yogurt during storage might be beneficial in lowering the flavor rating (Tamime et al., 1989). For instance, the addition of bioactive peptides to yogurt reduced odor ratings considerably (*p* < .05) compared with the control sample. On the first day of storage, treatment 1 (control) had the highest odor score, but there was no significant difference between the other treatments (Figure 2). On the eleventh day, the lowest odor score was associated with treatment 4 (yogurt containing 17 mg/mL of peptide), although no significant differences were seen between the other treatments (*p* < .05). On day 21, the treatment with the highest odor score was treatment 1 (control) and the treatment with the lowest odor score was treatment 4 (yogurt with 17 mg/mL of peptide). From the first to the twenty‐first day, the odor scores of treatments 1 (control), 2 (yogurt containing 6.5 mg/mL of peptide), and 3 (yogurt containing 13 mg/mL of peptide) did not vary substantially. From the first to the eleventh day, the odor score of treatment 4 (yogurt containing 17 mg/mL of peptide) decreased considerably (*p* < .05), whereas there was no significant change between the 11th and 21st days. According to Figure 2, there was no significant change in the texture scores of the samples on the first and eleventh days of storage. On the 21st day of storage, the greatest texture sensory assessment score was associated with treatment 4 (yogurt containing 17 mg/mL of peptide), and there was no significant difference between the other treatments. During storage, the texture score of samples containing bioactive peptides did not change. The rise in texture score in treatment 4 (yogurt containing 17 mg/mL of peptide) is due to the reduction in syneresis in this sample compared with other samples. During the storage period, there was no significant difference between treatments 1 (control), 2 (yogurt containing 6.5 mg/mL of peptide), and 3 (yogurt containing 13 mg/mL of peptide) in terms of color score and treatment 4 (yogurt containing 17 mg/mL of peptide) had the lowest color score. In fact, adding the maximum concentration of bioactive peptide to yogurt decreases color scores considerably (*p* < .05) compared with control yogurt and other samples. The drop in color score may be due to the influence of bioactive peptides on the casein network, which reduces the yogurt brightness index. The greatest overall acceptability sensory assessment score was associated with treatment 1 (control) on the first and eleventh days, whereas the lowest overall acceptability score was associated with treatment 4 (yogurt containing 17 mg/mL of peptide). On the 21st day of storage, treatment 4 (yogurt containing 17 mg/mL of peptide) had the lowest overall acceptability sensory assessment score, whereas there was no significant difference between the other treatments. During storage, there was no significant variation in the overall acceptability score of the samples. In fact, by adding bioactive peptides to yogurt, the overall acceptability ratings are considerably (*p* < .05) lowered, compared with those of the control yogurt. This is due to the loss of flavor, odor, and color scores in samples containing bioactive peptides. In a similar research, Bierzunska et al. (2019) found that taste ratings of yogurt samples containing hydrolyzed whey protein drop following 21 days of storage, but there is no significant difference between treatments in terms of odor scores. Texture ratings rose substantially compared with control yogurt; color scores did not drop significantly compared with control yogurt; and overall acceptability scores decreased significantly compared with control yogurt, which is consistent with the findings of this study. In another study, Oliveiralima et al. (2021) found that during 7 days of storage, odor and texture scores of stirred yogurt samples containing fish protein hydrolysate were significantly reduced compared with control yogurt, and overall acceptability scores were significantly lower than control yogurt. Moreover, in a similar investigation, Samadi Varedesara et al. (2021) found that during 15 days of storage, flavor, odor, texture, color, and overall acceptability scores of stirred yogurt samples containing grape seed protein hydrolysate increased compared with control yogurt.

## 4 CONCLUSION

The results of this study showed that all the peptide fractions isolated from soy whey protein hydrolysate had bioactive properties, but the F7 fraction indicated the highest antioxidant and antibacterial capabilities. Based on the results of enriching yogurt with various amounts of this peptide fraction, the antioxidant activity improves as the number of bioactive peptide increases, whereas viscosity and syneresis decrease. The acidity, syneresis, and viscosity of yogurt rose with storage, but the pH and antioxidant activity dropped. The addition of bioactive peptides reduced the number of pathogenic bacteria in yogurt during storage, and this reduction was stronger as the peptide content was increased. The sample containing the highest concentration of peptide had the lowest overall acceptance. The level of 13 mg/mL of peptide derived from soy whey was selected to be the ideal concentration for yogurt enrichment since other treatments did not vary substantially in terms of overall acceptance score. Therefore, this peptide can be used as a natural preservative and functional ingredient in yogurt.

## CONFLICT OF INTEREST STATEMENT

The authors declare that they have no conflict of interest.

## Data Availability

Research data are not shared.

## References

[fsn33312-bib-0001] Abadia‐Garcia, L. , Cardador, A. , del Campo, S. T. M. , Arvizu, S. M. , Castano‐Tostado, E. , Regalado‐Gonzalez, C. , Garcia‐Almendarez, B. , & Amaya‐Llano, S. L. (2013). Influence of probiotic strains added to cottage cheese on generation of potentially antioxidant peptides, anti‐Listerial activity and survival of probiotic, icroorganisms in simulated gastrointenstinal coditions. International Dairy Journal, 33(2), 191–197. 10.1016/j.idairyj.2013.04.005

[fsn33312-bib-0002] Aguilar Toala, J. E. , Santiago‐Lopez, L. , Peres, C. M. , Vallejo‐Cordoba, B. , Gonzalez‐Cordova, A. F. , & Hernandez‐Mendoza, A. (2017). Assessment of multi functional activity of bioactive peptides derived from fermented milk by specific *lactobacillus plantarum* strains. Journal of Dairy Science, 100(1), 65–75. 10.3168/jds.2016-11846 27865495

[fsn33312-bib-0003] Aleman, A. , Perez‐Santin, E. , Bordenavejuchereau, S. , Amaudin, I. , Gomez‐Guillen, M. C. , & Montero, P. (2011). Squid gelatin hydrolysate with anti hyper tensive, anti cancer and antioxidant activity. Food Research International, 44, 1044–1051. 10.1016/j.foodres.2011.03.010

[fsn33312-bib-0004] Altomare, A. A. , Baron, G. , Aldini, G. , Carini, M. , & D'Amato, A. (2020). Silkworm pupae as source of high‐value edible proteins and of bioactive peptides. Food Science & Nutrition, 8(6), 2652–2661. 10.1002/fsn3.1546 32566182 PMC7300080

[fsn33312-bib-0005] Anonymous . (2005). Iranian Institute of Standards and Industrial Research. Food Microbiology ‐ Counting Staphylococcus aureus. National Standard of Iran, No. 6806.

[fsn33312-bib-0006] Anonymous . (2015). Iranian Institute of Standards and Industrial Research. Milk and its products. Review of Escherichia coli. National Standard of Iran, No. 5234.

[fsn33312-bib-0007] AOAC . (2005). Official methods of analysis of the AOAC (18th ed., pp. 93–96). Association of Official Analytical Chemists.

[fsn33312-bib-0008] Aryana, K. J. , & McGrew, P. (2007). Quality attributes of yogurt with *lactobacillus casei* and various prebiotics. LWT‐Food Science and Technology, 40, 1808–1814. 10.1016/j.lwt.2007.01.008

[fsn33312-bib-0009] Bierzunska, P. , Sokolinska, D. , & Yigit, A. (2019). Storage stability of texture and sensory properties of yogurt with the addition of polymerized whey proteins. Journal of Health Sciences, 31, 600–624. 10.3390/foods8110548 PMC6915489 31689896

[fsn33312-bib-0010] Chang, O. , Han, S. , Seol, K. , Kim, H. , & Jeong, G. (2013). Novel antioxidant peptides derived from the ultrafiltrate of ovomucin hydrolysate. Journal of Agricultural and Food Chemistry, 61(30), 7294–7300. 10.1021/jf4013778 23834012

[fsn33312-bib-0011] Chumchuere, S. , MacDougall, D. B. , & Robinson, R. K. (2000). Production and properties of a semi‐hard cheese made from soyamilk. International Journal of Food Science and Technology, 35, 577–581. 10.1111/j.1365-2621.2000.00414.x

[fsn33312-bib-0012] Durand, E. , Beaubier, S. , Ilic, I. , Fine, F. , Kapel, R. , & Villeneuve, P. (2021). Production and antioxidant capacity of bioactive peptides from plant biomass to counteract lipid oxidation. Current Research in Food Science, 4, 365–397. 10.1016/j.crfs.2021.05.006 34142097 PMC8187438

[fsn33312-bib-0013] Ein Ali Afjeh, M. , Pourahmad, R. , Akbari Adergani, B. , & Azin, M. (2019). Use of glucose oxidase immobilized on magnetic chitosan nanoparticles in probiotic drinking yogurt. Food Science of Animal Resources, 39(1), 73–83. 10.5851/kosfa.2019.e5 30882076 PMC6411245

[fsn33312-bib-0014] Fang, T. , & Mingruo, G. (2019). Physicochemical, texture properties and micro structure of yogurt using polymerized whey protein directly prepared from cheese whey as a thichening agent. Journal of Dairy Science, 102(9), 7884–7894. 10.3168/jds.2018-16188 31301832

[fsn33312-bib-0015] Farzaneh, P. , Ehsani, M. R. , Khanahmadi, M. , & Sharifan, A. (2019). Characterization of bio‐peptides purified from *Terfezia claveryi* hydrolysate and their antibacterial effect on raw milk characterization of bio‐peptides purified from Terfezia claveryi hydrolysate and their antibacterial effect on raw milk. LWT‐Food Science and Technology, 116, 108522. 10.1016/j.lwt.2019.108522

[fsn33312-bib-0016] Ghaleh Mosiyani, Z. , Pourahmad, R. , & Eshaghi, M. R. (2017). Investigating the effect of aqueous extracts of basil and savory on antioxidant activity, microbial and sensory properties of probiotic yogurt. Acta Scientiarum Polonorum Technologia Alimentaria, 16(3), 311–320. 10.17306/J.AFS.0509 29055979

[fsn33312-bib-0017] Hang, Y. D. (2004). Management and utilization of food processing wastes. Journal of Food Science, 69(3), CRH104–CRH107. 10.1111/j.1365-2621.2004.tb13341.x

[fsn33312-bib-0018] Karami, Z. , & Akbari‐Adergani, B. (2019). Bioactive food derived peptides: A review on correlation between structure of bioactive peptides and their functional properties. Journal of Food Science and Technology, 56(2), 535–547. 10.1007/s13197-018-3549-4 30906011 PMC6400753

[fsn33312-bib-0019] Karimi, N. , Pourahmad, R. , Taheri, S. , & Eyvazzadeh, A. (2022). Investigation of the effect of adding bioactive peptide obtained by enzymatic hydrolysis of yogurt on the quality characteristics of doogh. Iranian Journal of Food Science and Technology, 18(4), 249–263. 10.22067/ifstrj.2106.1059

[fsn33312-bib-0020] Karimi, N. , Pourahmad, R. , Taheri, S. , & Eyvazzadeh, O. (2021). Isolation and purification of bioactive peptides from yogurt whey application as a natural preservation in a model food system. Journal of Food Processing and Preservation, 45(12), 307–319. 10.1111/jfpp.16086

[fsn33312-bib-0021] Kim, J. M. , Liceaga, A. M. , & Yoon, K. Y. (2019). Purification and identification of an antioxidant peptide from perilla seed (*Perilla frutescens*) meal protein hydrolysate. Food Science & Nutrition, 7(5), 1645–1655. 10.1002/fsn3.998 31139377 PMC6526660

[fsn33312-bib-0022] Liao, X. , Zho, Z. , Wu, S. , Chen, M. , Huang, R. , Wang, J. , Wu, Q. , & Ding, D. (2020). Preparation of antioxidant protein hydrolysates from Pleurotus geesteranus and their protective effects on H2O2 oxidative damaged PC12 cells. Molecules, 25, 5408. 10.3390/molecules25225408 33227951 PMC7699252

[fsn33312-bib-0023] Liu, X. , Zhao, M. , Wang, J. , Yang, B. , & Jiang, Y. (2008). Antioxidant activity of methanolic extract of emblica fruit (*Phyllanthus emblica L*.) from six regions in China. Journal of Food Composition and Analysis, 21, 219–228. 10.1016/j.jfca.2007.10.001

[fsn33312-bib-0024] Matemu, A. O. , Katayama, S. , Kayahara, H. , Murasawa, H. , & Nakamura, S. (2012). Improving surface functional properties of tofu whey‐derived peptides by chemical modification with fatty acids. Journal of Food Science, 77(4), C333–C339. 10.1111/j.1750-3841.2012.02631.x 22429318

[fsn33312-bib-0025] Mohan, N. M. , Zorgani, A. , Earley, A. , Chauhan, S. , Trajkovic, S. , Savage, J. , Adelfio, A. , Khaldi, N. , & Martins, M. (2019). Preservatives from food—For food: Pea protein hydrolysate as a novel bio‐preservative against *Escherichia coli* O157:H7 on a lettuce leaf. Food Science & Nutrition, 9(11), 5946–5958. 10.1002/fsn3.2489 PMC8565202 34760228

[fsn33312-bib-0026] Motta, A. S. , & Brandelli, A. (2002). Characterization of an antibacterial peptide produced by *Brevi*bacterium linens. Journal of Applied Microbiology, 92(1), 63–70. 10.1046/j.1365-2672.2002.01490.x 11849329

[fsn33312-bib-0027] Ni, H. , Hayes, E. , Stead, D. , & Raiko, S. V. (2018). Incorporating salal berry (*Gaultheria shallon*) black currant (*Ribes nigrum*) pomace in yogurt for the development of beverage with antidiabetic properties. Heliyon, 4(10), 1567–1573. 10.1016/j.heliyon.2018.e00875 PMC6205296 30386826

[fsn33312-bib-0028] Nicolas, P. (2009). Multifunctional host defence peptides: Intracellular targeting antimicrobial peptides. The FEBS Journal, 276(22), 6483–6496. 10.1111/j.1742-4658.2009.07359.x 19817856

[fsn33312-bib-0029] Oliveiralima, K. , Rocha, M. , Aleman, A. , Lopez‐Caballero, M. , Tover, C. , Geme Guillen, M. , & Moutero, P. (2021). Yogurt fortification by addition of microen capsulated stripped weak fish (Cynoscion guatucapa) protein hydrolysate. Antioxidants (Basel), 10(10), 1567–1573. 10.3390/antiox10101567 34679702 PMC8533301

[fsn33312-bib-0030] Pourahmad, R. , & Mazaheri Assadi, M. (2007). Use of isolated autochthonous starter cultures in yogurt production. International Journal of Dairy Technology, 60(4), 259–262. 10.1111/j.1471-0307.2007.00343.x

[fsn33312-bib-0031] Power, O. , Jakeman, P. , & Fitz Gerald, R. J. (2013). Antioxidative peptides: Enzymatic production in vitro and in vivo antioxidant activity and potential application of milk derived antioxidative peptidys. Amino Acids, 44, 797–820. 10.1007/s00726-012-1393-9 22968663

[fsn33312-bib-0032] Pownall, T. L. , Udenigwe, C. C. , & Aluko, R. E. (2010). Amino acid composition and antioxidant properties of pea seed (*Pisumsativum L*.) enzymatic protein hydrolysate fractions. Journal of Agricultural and Food Chemistry, 58, 4712–4718. 10.1021/jf904456r 20359226

[fsn33312-bib-0033] Saei‐Dehkordi, S. S. , Tajik, H. , Moradi, M. , & Khaleghi‐Sigaroodi, F. (2010). Chemical composition of essential oils in Zatoria multifloro biomass from different parts of Iran and their radical scavenging and antimicrobial activity. Food and Chemical Toxicolology, 48(6), 1562–1567. 10.1016/j.fct.2010.03.025 20332011

[fsn33312-bib-0034] Sah, B. N. P. , Vasiljevic, T. , McKechnie, S. , & Donkor, O. N. (2016). Physicochemical, textural and rheological properties of probiotic yogurt fortified with fibre‐rich pineapple peel powder during refrigerated storage. LWT‐Food Science and Technology, 65, 978–986. 10.1016/j.lwt.2015.09.027

[fsn33312-bib-0035] Sahan, N. , Yasar, K. , & Hayaloglu, A. A. (2008). Physical, chemical and flavour quality of non‐fat yogurt as affected by a β‐glucan hydrocolloidal composite during storage. Food Hydrocolloid, 22, 1291–1297. 10.1016/j.foodhyd.2007.06.010

[fsn33312-bib-0036] Samadi Varedesara, M. , Ariaii, P. , & Hesari, J. (2021). The effect of grape seed protein hydrolysate on the properties of stirred yogurt and viability of *lactobacillus casei* in it. Food Science & Nutrition, 9(4), 2180–2190. 10.1002/fsn3.2188 33841834 PMC8020923

[fsn33312-bib-0037] Sánchez, A. , & Vásquez, A. (2017). Bioactive peptides: A review. Food Quality and Safety, 1, 29–46. 10.1093/fqsafe/fyx006

[fsn33312-bib-0038] Shaik, M. I. , & Sarbon, N. M. (2020). A review on purification and characterization of anti‐proliferative peptides derived from fish protein hydrolysate. Food Reviews International, 38, 1389–1409. 10.1080/87559129.2020.1812634

[fsn33312-bib-0039] Shori, A. , Yong, Y. , & Baba, A. (2021). Effects of herbal yogurt with fish collagen on bioactive peptides with angiotensin‐I converting enzyme inhibitory activity. Journal of Food Science, 41(4), 185–188. 10.1590/fst.24020

[fsn33312-bib-0040] Singh, B. , Vij, P. , & Hati, S. (2014). Functional significance of bioactive peptides derived from soybean. Peptides, 54, 171–179. 10.1016/j.peptides.2014.01.022 24508378

[fsn33312-bib-0041] Tamime, A. Y. , Kalab, M. , & Davies, G. (1989). Rheology and microstructure of strained yogurt (labneh) made from cows milk by three different methods. Food Microstructure, 8, 125–135.

[fsn33312-bib-0042] Waili, Y. , Gahafu, Y. , Aobulitalifu, A. , Chang, Z. , Xie, X. , & Kawuli, G. (2021). Isolation, purification, and characterization of antioxidant peptides from fresh mare's milk. Food Science & Nutrition, 9(7), 4018–4027. 10.1002/fsn3.2292 34262755 PMC8269580

[fsn33312-bib-0043] Wang, R. , Zhao, H. , Pan, X. , Orfila, C. , Lu, W. , & Ma, Y. (2019). Preparation of bioactive peptides with antidiabetic, antihypertensive, and antioxidant activities and identification of α‐glucosidase inhibitory peptides from soy protein. Food Science & Nutrition, 7(5), 1848–1856. 10.1002/fsn3.1038 31139399 PMC6526634

[fsn33312-bib-0044] Wei, D. , & Zhang, X. (2022). Biosynthesis, bioactivity, biotoxicity and applications of antimicrobial peptides for human health. Biosafety and Heakth, 4, 118–134. 10.1016/j.bsheal.2022.02.003

[fsn33312-bib-0045] Wong, F. C. , Xiao, J. , Wang, S. , Ee, K. Y. , & Chai, T. T. (2020). Advances on the antioxidant peptides from edible plant sources. Trends in Food Science and Technology, 99, 44–57. 10.1016/j.tifs.2020.02.012

[fsn33312-bib-0046] Yong‐Chua, C. , & Shao‐Quan, L. (2019). Soy whey: More than just waste water from tofu and soy protein isolate industry. Trends in Food Science & Technology, 91, 1–36. 10.1016/J.TIFS.2019.06.016

